# Correlation of renal cortical blood perfusion and BP response after renal artery stenting

**DOI:** 10.3389/fcvm.2022.939519

**Published:** 2022-10-03

**Authors:** Siyu Wang, Sijie Zhang, Yan Li, Na Ma, Mengpu Li, Hu Ai, Hui Zhu, Junhong Ren, Yongjun Li, Peng Li

**Affiliations:** ^1^Department of Sonography, Beijing Hospital, National Center of Gerontology, Beijing, China; ^2^Institute of Geriatric Medicine, Chinese Academy of Medical Sciences, Beijing, China; ^3^Graduate School of Peking Union Medical College, Beijing, China; ^4^Department of Cardiology, Beijing Hospital, National Center of Gerontology, Beijing, China; ^5^Department of Nuclear Medicine, Beijing Hospital, National Center of Gerontology, Beijing, China; ^6^Department of Vascular Surgery, Beijing Hospital, National Center of Gerontology, Beijing, China; ^7^The Key Laboratory of Geriatrics, Beijing Institute of Geriatrics, Institute of Geriatric Medicine, Chinese Academy of Medical Sciences, Beijing Hospital/National Center of Gerontology of National Health Commission, Beijing, China

**Keywords:** renal artery stenosis, 24 h ambulatory blood pressure monitor, contrast-enhanced ultrasound, renal cortical blood perfusion, follow-up

## Abstract

**Background:**

This study aimed to observe the correlation between renal cortical blood perfusion (CBP) parameters and BP response in patients with severe renal artery stenosis (RAS) who underwent stenting.

**Methods:**

This was a single-center retrospective cohort study. A total of 164 patients with unilateral severe RAS after successful percutaneous transluminal renal artery stenting in Beijing Hospital from October 2017 to December 2020 were included. According to the results of BP evaluated at 12 months, all patients were divided into the BP response group (*n* = 98) and BP nonresponse group (*n* = 66). The baseline clinical and imaging characteristics and follow-up data about 24 h ABPM and CBP were recorded and analyzed. Pearson correlation analysis was used to evaluate the relationship between CBP parameters and 24 h average SBP. Univariate and multivariate logistic regression analysis was used to evaluate the risk factors for BP response.

**Results:**

Among 164 patients with severe RAS, there were 100 males (61.0%), aged 37–75 years, with an average of 56.8 ± 18.4 years, and average artery stenosis of 84.0 ± 12.5%. The BP nonresponse patients had a longer duration of hypertension, more current smoking subjects and diabetic patients, lower eGFR, increased number of hypertensive agents, and rate of insulin compared with the BP response group (*P* < 0.05). After PTRAS, patients in the BP response group were associated with significantly lower BP and improved CPB, characterized by increased levels of maximum intensity (IMAX), area under ascending curve (AUC1), area under the descending curve (AUC2), shortened rising time (RT), mean transit time (mTT), and prolonged time to peak intensity (TTP; *P* < 0.05). However, the BP nonresponse group was only associated with significantly reduced RT (*P* < 0.05) compared with baseline data. During an average follow-up of 11.5 ± 1.7 months, the BP response group was associated with significantly lower levels of SBP, DBP, 24 h average SBP, and 24 h average DBP compared with the nonresponse group (*P* < 0.05). Pearson correlation analysis showed that the the pre-operative CBP parameters, including IMAX (*r* = 0.317), RT (*r* = 0.249), AUC1 (*r* = 0.614), AUC2 (*r* = 0.558), and postoperative CBP parameters, including RT (*r* = 0.283), AUC1 (*r* = 0.659), and AUC2 (*r* = 0.674) were significantly positively correlated with the 24 h average SBP, while the postoperative TTP (*r* = −0.413) and mTT (*r* = −0.472) were negatively correlated with 24 h average SBP (*P* < 0.05). Multivariate Logistic regression analysis found that diabetes (OR = 1.294), NT-proBNP (OR = 1.395), number of antihypertensive agents (OR = 2.135), pre-operation IMAX (OR = 1.534), post-operation AUC2 (OR = 2.417), and baseline dDBP (OR = 2.038) were related factors for BP response (all *P* < 0.05).

**Conclusion:**

Patients in the BP nonresponse group often have diabetes, a longer duration of hypertension, significantly reduced glomerular filtration rate, and heavier renal artery stenosis. CBP parameters are closely related to 24 h average SBP, and pre-operation IMAX and post-operation AUC2 are markers for a positive BP response.

## Introduction

Renal artery stenosis (RAS) is associated with increased levels of BP. RAS is a primary cause of secondary hypertension. It involves the large and medium renal arteries, and is a relatively common condition in aged patients with hypertension, especially those with refractory hypertension, with the prevalence may be as high as 10–40% (1). RAS is conditioned mainly by atherosclerotic renal artery stenosis (ARAS), which primarily affects patients ≥45 years and usually involves the aortic artery orifice or the proximal main renal artery (2). In most cases of ARAS, ranging from 53 to 80%, one kidney is affected, with the main artery to the second kidney being essentially normal, hence the “unilateral” RAS (3). Percutaneous transluminal renal artery stenting (PTRAS) has emerged as the primary revascularization strategy in most patients with hemodynamically significant ARAS. However, several clinical randomized controlled trials, such as Cardiovascular Outcomes in Renal Atherosclerotic Lesions (CORAL) and Angioplasty and STent for Renal Artery Lesions, demonstrated that subjects with ARAS had similar outcomes whether randomized to optimal medical therapy alone or optimal medical therapy plus renal artery stenting (4, 5). In the CORAL study, after a median follow-up of 3.8 years, renal function was not improved after stenting; However, patients in the stented cohort were associated with a significant decrease in SBP (−14 mmHg) and DBP (−7 mmHg). Furthermore, the SBP and DBP changes were achieved with fewer antihypertensive drugs (−1 drug) (5).

There are several proven clinical predictors of BP response after renal artery stenting. Multiple single-center observational studies demonstrated that the renal artery stenting had a beneficial effect on BP response in 20–58% of patients (6, 7), and indicated several clinical factors for BP response, such as the number of antihypertensive drugs (OR = 5.9), preoperative DBP (OR = 13.9), clonidine use (OR = 4.5) (8), and brain natriuretic peptide (BNP) (*R* = 0.72) (9). In a retrospective study with 72 patients with resistant hypertension, Logistic regression analysis results showed that being younger (OR = 0.94), lower systolic daytime ambulatory BP (OR = 0.94), lower body mass index (OR = 0.82), and higher estimated glomerular filtration rate (eGFR) (OR = 2.73) were independent predictors for BP response after renal angioplasty (10). However, these markers had not been validated in prospective studies, and which patient obtains a positive BP response from renal artery stenting remains unclear.

Renal blood perfusion (CBP) may be associated with BP response after renal artery stent implantation. The kidney plays an important central role in the regulation of BP. Many experimental and physiological studies indicate that renal perfusion pressure is closely involved in maintaining BP (11). Renal artery perfusion pressure directly regulates sodium excretion and influences the activity of various vasoactive systems such as the renin-angiotensin-aldosterone (RAAS) system (12, 13). As a result, CBP assessed by contrast-enhanced ultrasound (CEUS) may be related to the BP response in renal artery stenting patients. Therefore, renal CBP parameters can be used as a marker of BP response. Our previous study showed that among patients with unilateral severe RAS who underwent stent implantation, those with improved CBP were associated with significantly improved BP control compared with patients with decreased CBP (*P* < 0.001) (14). However, there are few studies evaluating the relationship between renal CBP and BP response in RAS patients. Therefore, this study aimed to evaluate the relationship between CBP and BP response in RAS patients who underwent stent implantation.

## Methods

### Patients

This is a single-center retrospective cohort study. A total of 82 patients with unilateral severe ARAS after successful percutaneous transluminal renal artery stenting (PTRAS) in Beijing Hospital from October 2017 to December 2020 were included. There were 50 males (61.0%), aged 37–75 years, with an average artery stenosis of 84.0 ± 12.5%. This study has been registered in China Clinical Trial Registration Center (ChiCTR1800016252) and approved by the Institutional Review Board (IRB) of Beijing Hospital (No. 2018BJYYEC-043-02). Written consent was obtained for both the procedure and data collection in all cases.

### Inclusion and exclusion criteria

Inclusion criteria: (1) aged 18–80 years; (2) RAS was diagnosed by digital subtraction angiography (DSA), with unilateral atherosclerotic RAS of 70–99% and contralateral RAS < 50% (13, 14); (3) long diameter of the affected kidney >7 cm; (4) no residual stenosis or residual stenosis of <30% assessed by immediate post-operative DSA examination; and (5) with complete 12 months' follow-up data. Exclusion criteria: (1) unstable or severe cardiopulmonary dysfunction; (2) contrast agent allergy; (3) advanced tumors; and (4) poor CEUS images.

According to the results of APBM evaluated at 12 months, all patients were divided into the BP response group (*n* = 98) and BP nonresponse group (*n* = 66) (8). BP response was defined as a postoperative BP <160/90 mmHg with a less number of antihypertensive drugs or post-operative DB*P* < 90 mmHg with the same drugs. BP nonresponse was defined as maintaining the same antihypertensive drugs, or postoperative BP drops > 10 mmHg, with a reduced number and/or dose of antihypertensive drugs (8). The types and measurements of common antihypertensive drugs refer to the “Guidelines for the Prevention and Treatment of Hypertension in China (2018 Revised Edition” (15).

### Data collection

The patient's baseline characteristics, including age, gender, duration of hypertension and diabetes, and stenotic degree of renal artery were collected. In addition, routine kidney ultrasound examination parameters, such as kidney size, cortex thickness, and hemodynamic parameters, including the main renal artery peak systolic velocity (PSV), abdominal PSV, interlobar artery PSV, acceleration time, and resistance index, were recorded. Moreover, renal CBP' features before and after PTRAS were also recorded. All data were kept in the RAS Clinical and Imaging Database designed by Medical Research Statistics Center, Fuwai Hospital.

### RAS diagnoses

The current “gold standard” for RAS was digital subtraction angiography (DSA) and CEUS was used as a first-line screening method for evaluating RAS in our hospital (16). The Color Doppler ultrasound and CEUS examinations were performed with CA 1–7A (1–7 MHz) transducer on an RS80 ultrasound instrument (SAMSUNG, Korea). After routine renal artery ultrasonography, patients were injected with SonoVue (Sulfur Hexafluoride Microbubbles, Bracco, Milan, Italy) bolus two times into the upper limb vein for each kidney, including the main renal artery (dose, 1.0 ml/kidney) and renal CBP (dose, 1.2 ml/kidney) examination, followed by 5.0 ml saline for each bolus. First, patients were examined with normal breathing in the lateral position, and the dynamic contrast-enhanced renal artery imaging was stored for 1.0 min from the original site to the kidney hilum. The main renal artery lesion included the position, length, and diameter stenosis ratio. The degree of RAS was calculated as [1–(diameter of the stenosis/diameter of the normal portion distal to the stenosis)] ×100% in the artery phase of the enhanced image. And then the maximum long-axis section of the kidney was fixed to be perpendicular to the acoustic beam direction, SonoVue was injected again to continuously observe and store the real-time contrast agent perfusion of the renal cortical for 3 min (17, 18). Ultrasound instrument settings were kept constant during the entire procedure, including the contrast mechanical index MI of 0.08, the image depth of 14 cm, and the gain of 60 dB. The interval between each contrast agent injection was 15 min.

### CBP Assessment

**Figure 1:**
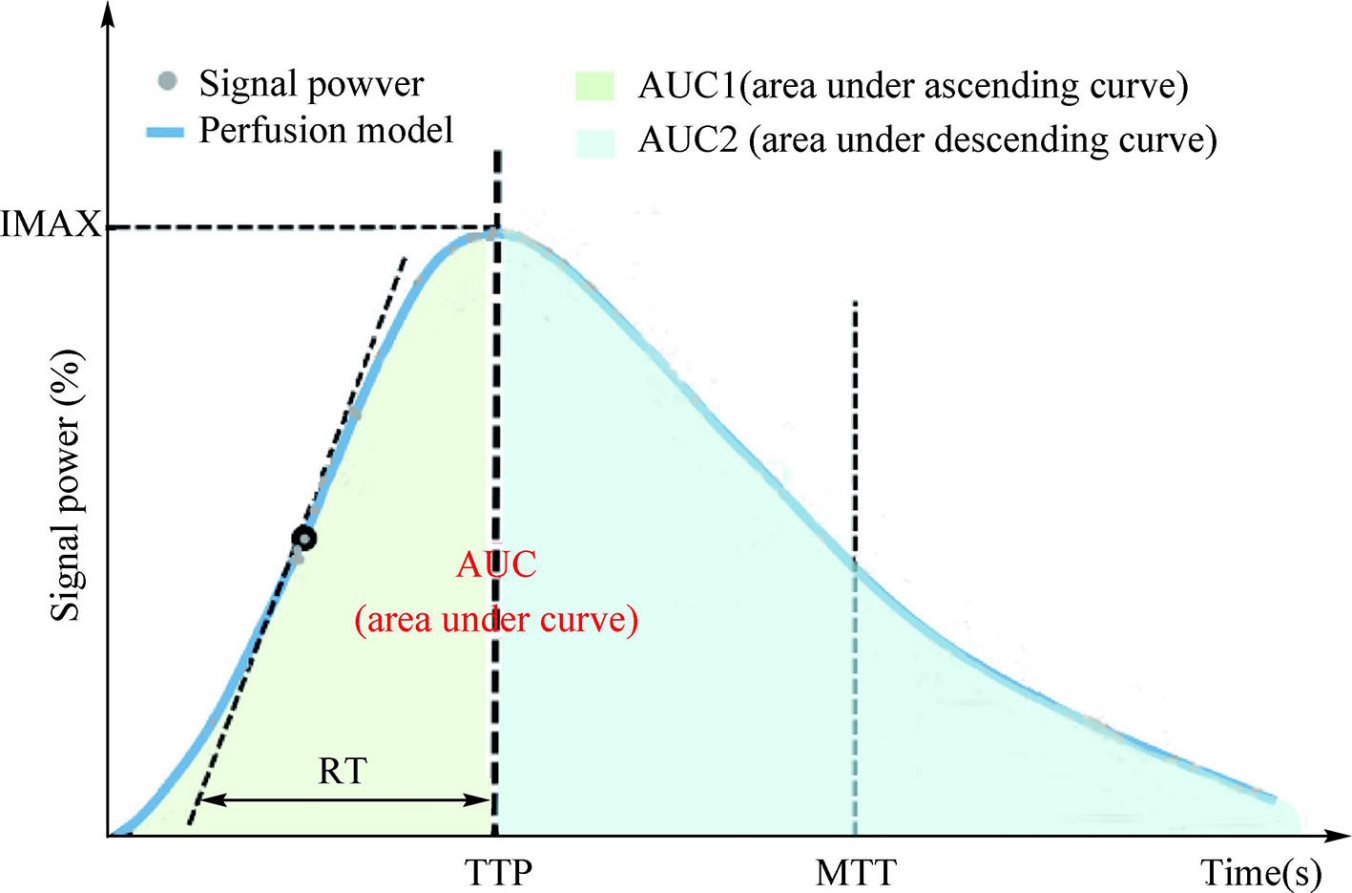
Time-dependent intensity curves based on selected regions of interest.

After all the examination procedures, the time-intensity curve (TIC) of renal cortical regions of interest (ROI) was analyzed using TomTec Imaging Systems (Germany) to determine the parameters of renal cortical microvascular perfusion, including the area under ascending curve (AUC1), area under the descending curve (AUC2), rising time (RT), time to peak intensity (TTP), maximum intensity (IMAX, with respect to the IMAX of the reference ROI), and mean transit time (MTT; Figure 1). Experts from the Departments of Sonography (Na Ma, Jun-Hong Ren), Vascular Surgery (Yong-Jun Li), and Cardiology (Hu Ai) independently determined the RAS diagnoses, and two experienced sonographers (Na Ma and Jun-Hong Ren) reviewed the CBP.

### 24 h ambulatory BP monitoring

All patients wore an ABPM-05 ambulatory BP monitor (ABPM; Merrill Lynch, USA) on their left upper arm for 24 h, and kept the sea level at rest during each measurement. From 6:00 to 22:00, the day-time BP will be measured every 20 min, and between 22:00 and 6:00 the next day, the night-time BP will be measured every 1 h. And the following are recorded: 24 h average SBP, 24 h average DBP, daytime systolic BP (dSBP), dDBP, nighttime systolic BP (nSBP), and nDBP.

### Statistical analyses

Data analysis was performed through STATA 13.0 statistical software (version 14.0; StataCorp, College Station, TX, USA). Normal distribution of measurement data was expressed as mean and SD, comparison between groups was analyzed by *t*-test or one-way ANOVA; non-normally distributed measurement data were represented by median (interquartile range) and non-parametric tests were used for comparison between groups; countable data were expressed as percentages and comparisons between groups were detected by the χ^2^ test. The Pearson correlation analysis was used to evaluate the relationship between pre- or post-operative CBP parameters and 24 h average SBP measured at 12 months of follow-up. *P* < 0.05 was considered statistically significant.

## Results

### Baseline data comparison between the two groups

The BP nonresponse group had a longer duration of hypertension, more current smoking subjects and diabetic patients, lower eGFR, higher NT-proBNP, increased number of hypertensive agents, and rate of insulin compared with the BP response group (all *P* < 0.05) (Table 1).

**Table 1:** Comparison of the baseline data of the study groups.

**Characteristics**	**Nonresponse group (*n* = 66)**	**Response group (*n* = 98)**	***t*** **/*Z*/χ^2^ value**	***P*** **-value**
Baseline data				
Age, yr	55.6 ± 14.7	54.3 ± 12.8	0.291	0.771
Male, *n* (%)	38 (57.6)	62 (63.3)	0.5337	0.464
Current smoking, *n* (%)	42 (63.6)	42 (42.9)	6.816	0.009
BMI, kg/m^2^	26.1 ± 3.4	26.4 ± 3.1	0.584	0.560
Duration of HP, yr	10.9 ± 4.2	7.3 ± 2.2	7.158	0.001
Previous history				
Diabetes, *n* (%)	40 (60.6)	46 (46.9)	0.954	0.046
Hyperlipidemia, *n* (%)	44 (66.7)	64 (65.3)	0.033	0.857
OMI, *n* (%)	32 (48.5)	40 (40.8)	0.942	0.332
Atrial fibrillation, *n* (%)	28 (42.4)	36 (36.7)	0.537	0.464
Lab. test				
eGFR(ml/min/1.73m^2^)	50.7 ± 15.7	56.7 ± 13.9	2.572	0.011
NT-proBNP	379.2 ± 203.1	327.4 ± 154.9	1.951	0.033
LVEF, %	47.3 (38.4,58.6)	49.7 (43.7,55.2)	0.625	0.533
Degree of RAS, %	81.4 ± 11.9	80.8 ± 10.2	0.345	0.731
Drug treatment				
Antihypertensive agents, *n*	4.3 ± 1.7	3.8 ± 1.8	0.834	0.038
CCB^§^	65 (98.5)	98 (100)	1.494	0.222
β-blocker	26 (39.4)	32 (32.7)	0.874	0.376
ACEI/ARB	13 (19.7)	20 (20.4)	0.012	0.911
Diuretic	22 (33.3)	21 (21.4)	2.889	0.089
Statins, *n* (%)	36 (54.5)	50 (51.0)	0.197	0.658
Insulin, *n* (%)	28 (42.4)	28 (28.6)	3.367	0.047
Antiplatelet therapy, *n* (%)	44 (66.7)	64 (65.3)	0.033	0.857
Anticoagulant, *n* (%)	26 (39.4)	34 (34.7)	0.376	0.540

§ CCB included amlodipine, L-amlodipine, nifedipine, felodipine, lercanidipine; BMI, body mass index; HP, hypertension; OMI, old myocardial infarction; Scr, serum creatinine; eGFR, estimated glomerular filtration rate; LVEF, left ventricular ejection fraction; RAS, renal artery stenosis; CCB, calcium channel blocker; ACEI, angiotensin-converting enzyme inhibitor; ARB, angiotensin receptor blocker.

### Pre- and post-operative renal CBP data

Before stent implantation, the CBP of the two groups was significantly different (*P* < 0.05). Compared with the BP nonresponse group, the response group had better cortical perfusion, which was manifested as a significant decrease in IMAX, a shortened RT and TTP time, a prolonged mTT time, and a significant increase in AUC1 and AUC2 (*P* < 0.05).

**Figure 2:**
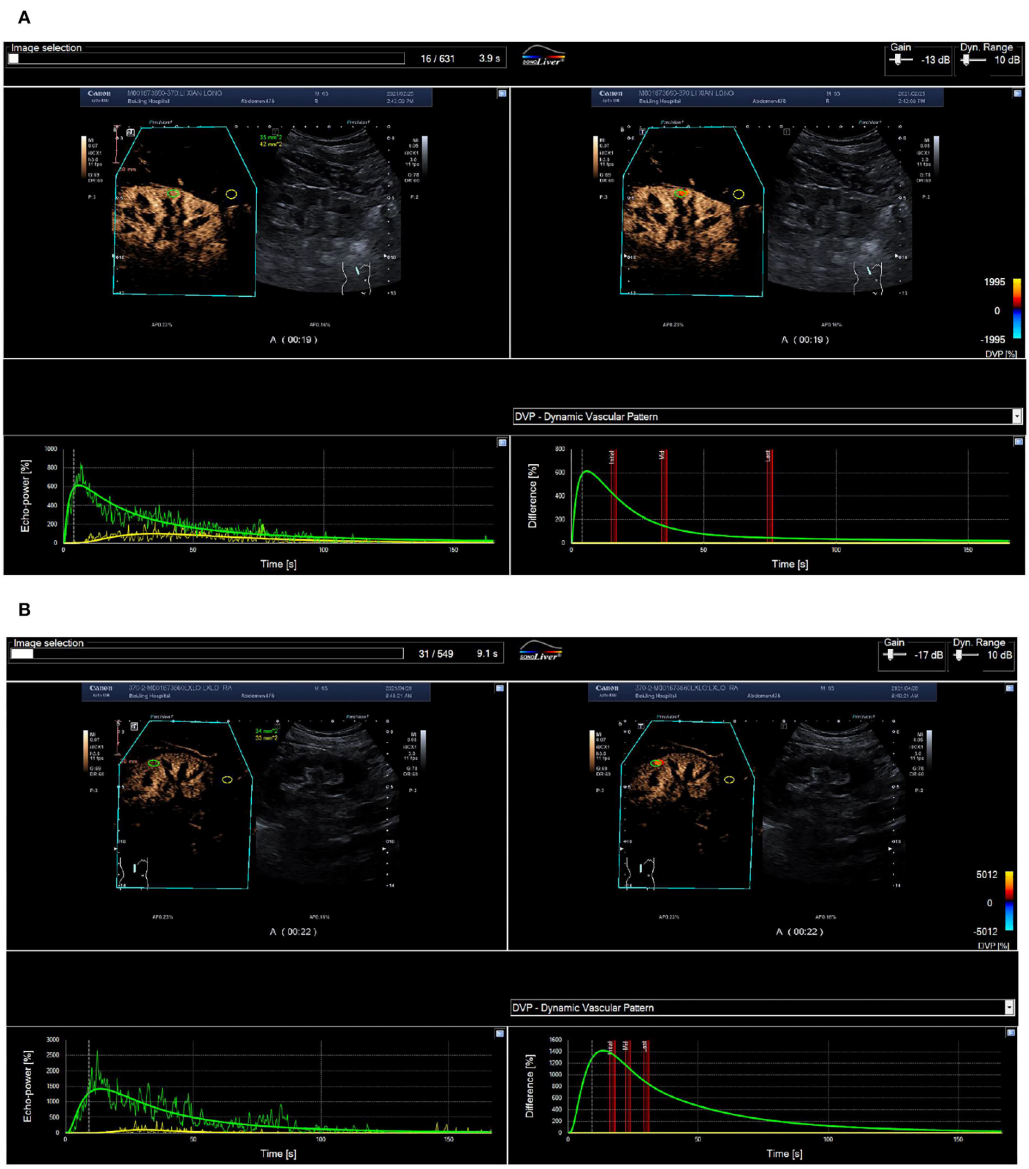
Routine ultrasound and CEUS images of a 65-year-old man with 70% right renal ostial stenosis. **(A)** Color Doppler flow (left), Doppler frequency spectrum (middle), and CEUS (right) images of the long axis section of right renal artery before PTRAS. **(B)** After stent implantation, renal artery blood flow images (left) and the peak systolic velocity (middle) of stenosis were corrected, and contrast beam filling (right) displayed normal. RA, renal artery; AO, abdominal aorta. **(A)** Pre-operative cortical blood perfusion, IMAX (%): 855.1, RT (s): 7.7, TTP (s): 8.3, mTT (s): 42.5, AUC1: 47.5, AUC2: 308.4; **(B)** Post-operative cortical blood perfusion, IMAX (%): 725.3, RT (s): 6.7, TTP (s): 7.3, mTT (s): 27.9, AUC1: 34.6, AUC2: 194.4. Changes in cortical blood perfusion before **(A)** and after **(B)** stent implantation of a 57-year-old woman with severe stenosis of the right renal artery in the BP nonresponse group.

After stent implantation, the BP nonresponse group was associated with significantly reduced RT (*P* < 0.05), but other CBP parameters (IMAX, TTP, mTT, AUC1, and AUC2) did not change significantly (*P* > 0.05) (Figure 2). In the BP response group, IMAX, AUC1, and AUC2 were significantly increased, while the RT and mTT were significantly shortened (*P* < 0.05), and TTP was significantly prolonged compared with pre-operation (*P* < 0.05) (Table 2).

**Table 2:** Cortical blood perfusion parameters before and after stent implantation.

**Parameters**	**Pre/post-operation**	**Nonresponse group (*n* = 66)**	**Response group (*n* = 98)**	***t*** **-value**	***P*** **-value**
Imax (%)	Pre-	765.4 ± 224.7	717.2 ± 222.6	1.355	0.177
	Post-	875.3 ± 231.2	1242.7 ± 154.6	12.202	0.001
	*t*-value	2.769	19.195		
	*P*-value	0.006	0.0001		
RT(s)	Pre-	6.6 ± 2.2	5.8 ± 1.7	2.622	0.011
	Post-	6.0 ± 1.5	11.9 ± 2.2	19.005	0.0001
	*t*-value	1.831	21.719		
	*P*-value	0.035	0.0001		
TTP(s)	Pre-	6.4 ± 3.4	5.8 ± 1.7	1.493	0.137
	Post-	7.2 ± 2.7	12.9 ± 3.2	11.895	0.0001
	*t*-value	1.497	19.397		
	*P*-value	0.137	0.0001		
mTT(s)	Pre-	46.3 ± 27.4	59.6 ± 21.2	3.497	0.001
	Post-	39.8 ± 23.5	48.4 ± 17.6	2.767	0.008
	*t*-value	1.463	4.024		
	*P*-value	0.146	0.001		
AUC1(dB × s)	Pre-	50.2 ± 27.4	61.3 ± 26.4	2.601	0.010
	Post-	64.3 ± 25.6	103.4 ± 33.7	7.996	0.001
	*t*-value	3.055	9.735		
	*P*-value	0.003	0.0001		
AUC2(dB × s)	Pre-	247.3 ± 179.4	275.5 ± 150.3	1.089	0.278
	Post-	337.4 ± 153.1	563.2 ± 202.5	7.695	0.001
*t*-value	*t*-value	3.104	11.294		
	*P*-value	0.002	0.0001		

**Figure 3:**
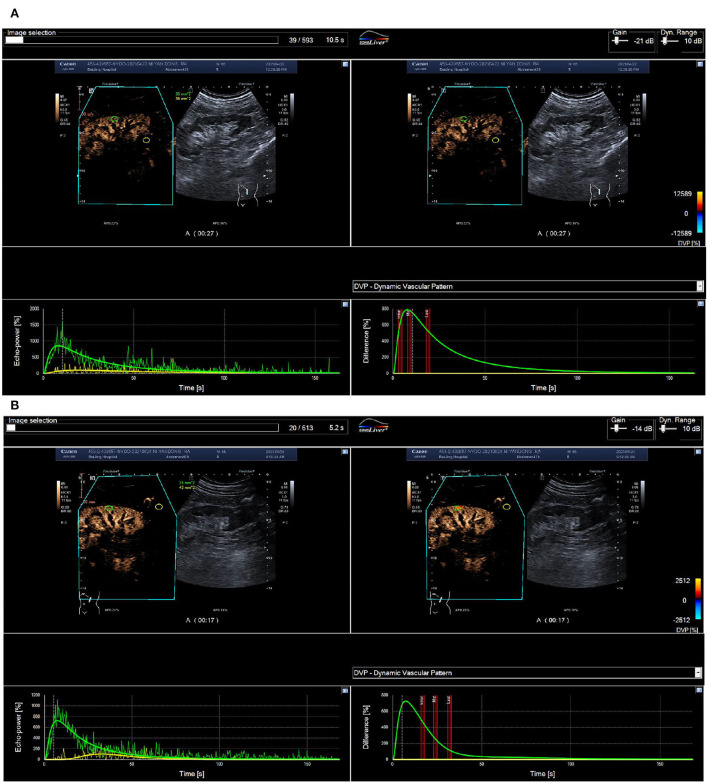
ROC curve of renal blood perfusion parameters for predicting poor prognosis. **(A)** Pre-operative cortical blood perfusion, IMAX (%): 614.7, RT (s): 5.8, TTP (s): 5.9, mTT (s): 78.4, AUC1: 26.0, AUC2: 251.6; **(B)** Post-operative cortical blood perfusion, IMAX (%): 1422.4, RT (s): 12.2, TTP (s): 13.6, mTT (s): 45.6, AUC1: 119.4, AUC2: 632.6. Changes in cortical blood perfusion before **(A)** and after **(B)** stent implantation of a 53-year-old man with severe stenosis of the left renal artery in the BP response group.

After stent implantation, compared with the BP nonresponse group, the CBP of the BP response group was significantly different, manifested as a significant increase in IMAX, AUC1, and AUC2, and an increase in RT, mTT, and TTP (*P* < 0.05) (Figures 2, 3).

### Results of ABPM at 12-month follow-up

The baseline BP levelsof the two groups were similar, and there were no significant differences in dSBP, nSBP, and 24 h average SBP (*P* > 0.05). However, levels of dDBP, nDBP, and 24 h average DBP were significantly higher in the response group (*P* < 0.05).

At the 12-month follow-up, compared with baseline level, the BP nonresponse group was associated with significantly reduced dSBP, nSBP, and 24 h average SBP (*P* < 0.05), but dDBP, nDBP, and 24 h average DBP did not change significantly (*P* > 0.05).

The BP response group was associated with significantly reduced SBP, nSBP, nDBP, 24 h average SBP, and 24 h average DBP (*P* < 0.05), but the levels of dDBP did not change significantly (*P* > 0.05). Additionally, at 12 months of follow-up, compared with the nonresponse group, the response group was associated with significantly lower levels of dSBP, dDBP, nSBP, nDBP, 24 h average SBP, and 24 h average DBP (*P* < 0.05) (Table 3).

**Table 3:** The 24 h ABP at baseline and 12 months of follow-up between groups.

**BP**	**Time**	**Nonresponse group (*n* = 66)**	**Response group (*n* = 98)**	***t*** **-value**	***P*** **-value**
dSBP	Baseline	154.2 ± 11.4	153.9 ± 11.6	0.164	0.870
	Follow up	139.1 ± 9.7	135.1 ± 8.4	2.809	0.006
	*t*-value	8.196	12.995		
	*P*-value	0.0001	0.0001		
dDBP	Baseline	89.2 ± 10.2	91.2 ± 11.1	1.169	0.244
	follow-up	86.4 ± 6.3	84.7 ± 8.6	1.376	0.171
	*t*-value	1.897	4.583		
	*P*-value	0.061	0.001		
nSBP	Baseline	149.4 ± 9.7	150.3 ± 12.3	0.499	0.619
	follow-up	137.5 ± 8.1	131.4 ± 5.8	5.620	0.001
	*t*-value	7.651	13.759		
	*P*-value	0.0001	0.0001		
nDBP	Baseline	88.4 ± 11.3	89.5 ± 13.9	0.545	0.594
	follow-up	85.2 ± 5.9	82.6 ± 6.2	2.685	0.008
	*t*-value	2.039	4.488		
	*P*-value	0.043	0.001		
24 h SBP	Baseline	151.3 ± 9.2	151.6 ± 12.7	0.165	0.869
	follow-up	140.3 ± 6.7	135.3 ± 5.1	5.418	0.001
	*t*-value	7.852	11.791		
	*P*-value	0.0001	0.0001		
24 h DBP	Baseline	88.7 ± 11.8	90.1 ± 12.4	0.723	0.471
	follow-up	85.3 ± 8.4	83.2 ± 7.3	1.701	0.046
	*t*-value	1.907	4.747		
	*P*-value	0.058	0.001		

### Correlation of CBP and 24 h average SBP

**Figure 4:**
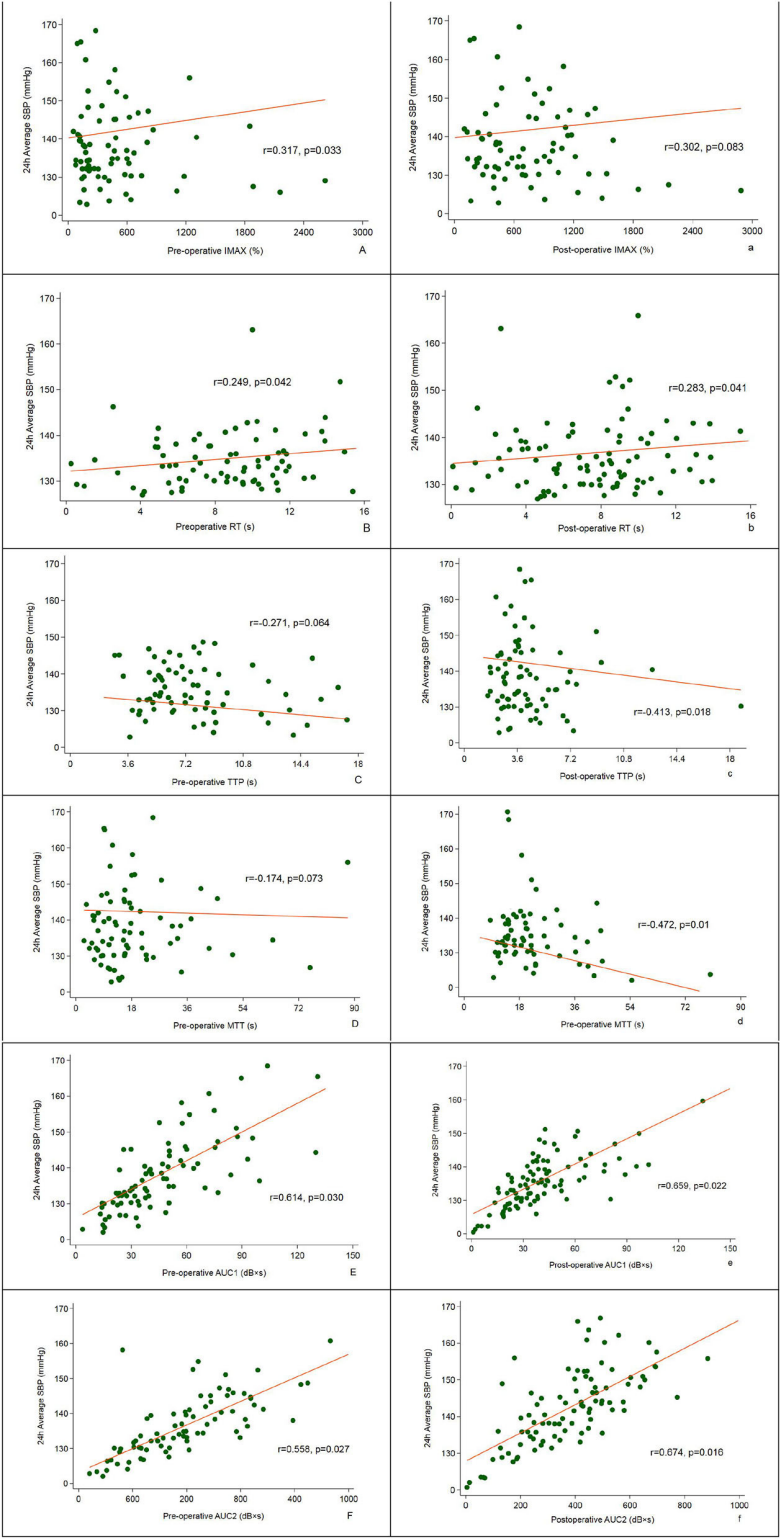
Correlation between pre- and post-operative CBP and 24 h average SBP assessed at 12-month follow-up.

As shown in Figure 3, the pre-operative CBP parameters, IMAX (*r* = 0.317), RT (*r* = 0.249), AUC1 (*r* = 0.614), and AUC2 (*r* = 0.558) were all significantly positively correlated with the 24 h average SBP at follow-up; Postoperative CBP parameters, RT (*r* = 0.283), AUC1 (*r* = 0.659), and AUC2 (*r* = 0.674) were significantly positively correlated with the 24 h average SBP, and TTP (*r* = −0.413) and mTT (*r* = −0.472) were negatively correlated with 24 h average SBP (*P* < 0.05) (Figure 4).

### Univariate and multivariate logistic regression analysis

Univariate Logistic regression analysis showed that duration of hypertension (OR = 2.091), smoking (OR = 1.628), diabetes (OR = 2.736), eGFR (OR = 1.954), NT-proBNP (OR = 3.703), number of antihypertensive agents (OR = 4.189), pre-operation IMAX (OR = 2.872), post-operation RT (OR = 1.827), post-operation AUC1 (OR = 3.554), post-operation AUC2 (OR = 4.872), baseline dDBP (OR = 3.703), and baseline nDBP (OR = 2.412) were risk factors for BP response (all *P* < 0.05).

Multivariate Logistic regression analysis found that diabetes (OR = 1.294), NT-proBNP (OR = 1.395), number of antihypertensive agents (OR = 2.135), pre-operation IMAX (OR = 1.534), post-operation AUC2 (OR = 2.417), and baseline dDBP (OR = 2.038) were related factors for BP response (all *P* < 0.05) (Table 4).

**Table 4:** Univariate and multivariate Logistic regression analysis.

**Risk factor**	**Univariate**			**Multivariate**		
	**OR**	**95% CI**	***P*** **-value**	**OR**	**95% CI**	***P*** **-value**
Duration of HP	2.091	1.287–3.397	0.003	—	—	—
Smoking	1.628	1.153–2.299	0.006	—	—	—
Diabetes	2.736	1.554–4.817	0.0005	1.294	1.013–1.628	0.039
eGFR	1.954	1.176–3.247	0.010	—	—	—
NT-proBNP	3.703	1.584–8.657	0.003	1.395	1.115–1.745	0.004
Number of antihypertensive agents	4.189	2.072–8.469	0.0001	2.135	1.236–3.688	0.007
Pre-operation IMAX	2.872	1.381–5.973	0.005	1.534	1.112–2.116	0.009
Post-operation RT	1.827	1.229–2.716	0.003	–	–	–
Post-operation AUC1	3.554	1.503–8.404	0.004	1.627	1.143–2.316	0.007
Post-operation AUC2	4.872	2.779–13.343	0.002	2.417	1.295–4.188	0.002
Baseline dDBP	3.703	1.282–10.696	0.016	2.038	1.296–3.305	0.002
Baseline nDBP	2.412	1.271–4.577	0.007	–	–	–

## Discussion

In our study, patients in the BP nonresponse group often have diabetes, a longer duration of hypertension, significantly reduced glomerular filtration rate, and heavier renal artery stenosis. CBP parameters, especially postoperative AUC1 and AUC2, are positively related to 24 h average SBP, and IMAX and AUC2 are positively related to BP response.

Previous studies indicated several clinical and laboratory markers for BP response in RAS patients. Modrall and its colleagues enrolled 149 patients who underwent primary renal artery stenting. Patients were divided into the “responders group” and nonresponder group. BP responders were defined based on modified AHA guidelines: BP <160/90 mmHg with fewer antihypertensive drugs or DBP <90 mmHg with the same drugs. All others were regarded as “nonresponders.” After a median follow-up of 19 months, the study indicated that a favorable BP response rate was 34%. Furthermore, three independent predictors of a positive BP response were identified: (1) with four or more antihypertensive drugs (OR = 29.9, *P* = 0.0001), (2) preoperative DBP >90 mmHg (OR = 31.4, *P* = 0.0011), and (3) with preoperative clonidine use (OR = 7.3, *P* = 0.029) (8). In addition, among subjects with three antihypertensive drugs, those with ipsilateral kidney volume ≥ 150 cm^3^ had a 3-fold increased BP response rate compared with patients with smaller kidneys (*P* = 0.018) (8). However, that study's sample size was small. Moreover, those markers for BP response have not been independently validated in other studies (19). Subsequently, they conducted another study to validate these markers using the CORAL trial data. There were 436 subjects who underwent stent implantation in the CORAL trial. Baseline SBP and DBP were 149 mmHg and 78 mmHg in the stented cohort, respectively (19). Multilogistic regression analysis results confirmed the three independent markers for BP response, including ≥4 antihypertensive drugs (OR = 5.9, *P* < 0.001), preoperative DBP >90 mmHg (OR = 13.9, *P* < 0.001), and preoperative clonidine use (OR = 4.52, *P* = 0.008) (19). Meanwhile, the BP response rate increased incrementally with the increase in the number of markers (*P* < 0.0001) (19). In another study conducted by Silva et al. 27 RAS patients who underwent stent placement were enrolled. The authors found that BNP was elevated before stenting (187 pg/ml) and fell within 24 h after stenting (median, 96 pg/ml), and remained low (85 pg/ml) at follow-up (20). Furthermore, they showed that patients with a higher baseline BNP (>80 pg/ml) had a higher rate of BP improvement than those with lower BNP (≤ 80 pg/ml) (20). In contrast with this study, Jaff et al. indicated no correlation between BP response and baseline BNP (21). Meanwhile, in a patient-level meta-analysis of 901 subjects from five prospective studies, Weinberg et al. found that elevated baseline SBP of more than 150 mmHg was also significantly associated with BP response (OR = 1.76) (22). Prajapati et al. also showed that male gender (*P* = 0.016), higher baseline SBP (*P* < 0.01), DBP (*P* < 0.04), no. of antihypertensive drugs (*P* < 0.01), and low GFR < 60 ml/min (*P* < 0.01) were correlated with poor BP response (23). Rocha-Singh et al. identified the preoperative MAP of >110 mmHg (OR = 2.90) and bilateral renal stenoses (OR = 4.60) as markers of BP response (24). In line with previous studies (25), we confirmed that diabetes (OR = 3.294), number of antihypertensive agents (OR = 7.485), NT-proBNP (OR = 2.395), and dDBP (OR = 9.238) were markers of BP response.

Recently, several studies proved that CBP was considered to be a sensitive indicator for BP response after stent implantation. In a small-sample observational study involving 68 significant RAS (≥70% stenosis) patients, who underwent stenting and with refractory hypertension (BP ≥ 140/90 mmHg despite therapy with two or more antihypertensive drugs), the authors evaluated the renal perfusion with renal frame count (RFC; angiographic frame number for contrast to reach distal renal parenchyma after initial renal artery opacification) and changes of BP (baseline and at 6-month follow-up) were recorded (26). The primary outcome was the clinical response, which was defined as a reduction in SBP >10 mmHg with the same or fewer antihypertensive drugs. The study indicated that subjects with RFC > 30 had significant reductions in SBP, DBP, and MAP (*P* < 0.05). Moreover, those with baseline RFC > 30 was associated with a significantly increased rate of clinical response to stenting (93.5 vs. 73.0%, *P* = 0.027) (26). Another subsequent trial confirmed these findings and showed that BP responders had a greater decrease in RFC and 78.6% of subjects with >4 RFC decrease were BP responders (*P* = 0.024) (27). Therefore, renal perfusion was a marker for BP response. Besides, renal artery pressure-related index was also associated with BP response. Mangiacapra and its colleagues enrolled 53 consecutive hypertensive subjects with unilateral RAS and evaluated the translesional pressure gradient (TPG). After a mean follow-up of 3 months, they found that dopamine-induced TPG (≥20 mmHg) was the only marker of BP response (AUC, 0.77; 95% CI 0.64–0.90) (28). In another study, Leesar et al. compared the predictive values of TPG [resting systolic gradient, hyperemic systolic gradient (HSG), fractional flow reserve (FFR), mean gradient], intravascular ultrasound (IVUS), and angiographic index for hypertension improvement. They found that compared with FFR (AUC = 0.85), IVUS area stenosis (AUC = 0.82) and diameter stenosis by angiography (AUC = 0.74), HSG (AUC = 0.74) was the best marker for hypertension improvement. Furthermore, multivariate logistic regression analysis indicated that HSG was the only independent parameter for predicting BP response (OR = 1.39, 95% CI 1.05–1.65) (29). However, Mitchell et al. found the opposite results. In a prospective study that only enrolled 17 unilateral RAS patients, they showed that TPG parameters (resting, peak, or hyperemic) alone could not differentiate BP responders, and renal FFR was a marker for BP response (30). However, this study's sample size is small and patients were only followed for 90 d. Therefore, it could not completely exclude the results of other studies. The AHA panel recommends that a peak systolic TPG ≥ 20 mmHg, or mean gradient ≥10 mmHg, is the indicator for revascularization in patients with symptomatic RAS. In our study, after an average follow-up of 11.5 months, the BP response group was associated with significantly lower levels of SBP, DBP, 24 h average SBP, and 24 h average DBP compared with the nonresponse group (*P* < 0.05). Pearson correlation analysis showed that the the pre-operative CBP parameters, including IMAX (*r* = 0.317), RT (*r* = 0.249), AUC1 (*r* = 0.614), and AUC2 (*r* = 0.558), and postoperative CBP parameters, including RT (*r* = 0.283), AUC1 (*r* = 0.659), and AUC2 (*r* = 0.674), were significantly positively correlated with the 24 h average SBP, while the postoperative TTP (*r* = −0.413) and mTT (*r* = −0.472) were negatively correlated with 24 h average SBP (*P* < 0.05). Furthermore, multivariate Logistic regression analysis demonstrated that diabetes (OR = 1.294), NT-proBNP (OR = 1.395), number of antihypertensive agents (OR = 2.135), pre-operation IMAX (OR = 1.534), post-operation AUC2 (OR = 2.417), and baseline dDBP (OR = 2.038) were related factors for BP response (all *P* < 0.05). AUC2 is the index for renal perfusion and IMAX is closely related to renal artery pressure. Therefore, RBP parameters, preoperative IMAX, and postoperation AUC2 are makers of a positive BP response. However, we did not assess the TPG, FFR, and IVUS area stenosis and could evaluate the predictive value for BP response among these parameters.

## Limitations

This study had some limitations. (1) This study was a single-center cohort with a small sample. (2) All patients included in our study had atherosclerotic RAS, and those with a non-atherosclerotic reason of RAS, such as Takayasu's arteritis and fibromuscular dysplasia, may have different characteristics, rate of BP response, and its related factors (31). (3) Patients enrolled were often middle-aged and elderly and had several atherosclerotic-related factors. Therefore, those younger patients with few atherosclerotic-related factors may have different related factors for renal function deterioration (31). (4) In the clinic, more than 1/2 of moderate-to-severe RAS patients had bilateral lesions and both kidneys were related to BP response. However, patients included in our study had unilateral RAS (31). (5) In addition, longer follow-up data are needed to evaluate the prognosis (32). More high-quality studies with large sample size and long follow-ups are warranted to validate these findings (33).

## Conclusion

In conclusion, patients in the BP nonresponse group often have diabetes, a longer duration of hypertension, significantly reduced glomerular filtration rate, and heavier renal artery stenosis. CBP parameters are closely related to 24 h average SBP, and pre-operation IMAX and post-operation AUC2 are markers for a positive BP response. However, more studies are warranted to validate these findings.

## Data availability statement

The raw data supporting the conclusions of this article will be made available from the corresponding author by request.

## Ethics statement

The studies involving human participants were reviewed and approved by the Beijing Hospital. The patients/participants provided their written informed consent to participate in this study.

## Author contributions

SW and SZ wrote the main manuscript text. YanL, NM, ML, HA, HZ, and PL analyzed the data. JR designed this work. All authors contributed to the article and approved the submitted version.

## Funding

This study was funded by the National High Level Hospital Clinical Research Funding (No. BJ-2018-198), Basic Research Project of the Central Academy of Medical Sciences of China (No. 2019PT320012), Beijing Science and Technology Project (No. Z211100002921011), and National Key R&D Program of China (No. 2020YFC2008100).

## Conflict of interest

The authors declare that the research was conducted in the absence of any commercial or financial relationships that could be construed as a potential conflict of interest.

## Publisher's note

All claims expressed in this article are solely those of the authors and do not necessarily represent those of their affiliated organizations, or those of the publisher, the editors and the reviewers. Any product that may be evaluated in this article, or claim that may be made by its manufacturer, is not guaranteed or endorsed by the publisher.

## References

[1] Safian RD. Renal artery stenosis. Prog Cardiovasc Dis. (2021) 65:60–70. 10.1016/j.pcad.2021.03.003 33745915

[2] Tafur JD White CJ. Renal artery stenosis: when to revascularize in 2017. Curr Probl Cardiol. (2017) 42:110–35. 10.1016/j.cpcardiol.2017.01.004 28325353

[3] Prince M Tafur JD White CJ. When and how should we revascularize patients with atherosclerotic renal artery stenosis? JACC Cardiovasc Interv. (2019) 12:505–17. 10.1016/j.jcin.2018.10.023 30898248

[4] Investigator ASTRAL Wheatley K Ives N Gray R Kalra PA Moss JG. Revascularization versus medical therapy for renal-artery stenosis. N Engl J Med. (2009) 361:1953–62. 10.1056/NEJMoa0905368 19907042

[5] Cooper CJ Murphy TP Cutlip DEK Henrich W Reid DM. Stenting and medical therapy for atherosclerotic renal-artery stenosis. N Engl J Med. (2014) 370:13–22. 10.1056/NEJMoa1310753 24245566 PMC4815927

[6] Nolan BW Schermerhorn ML Rowell E Powell RJ Fillinger MF Rzucidlo EM. Outcomes of renal artery angioplasty and stenting using low-profile systems. J Vasc Surg. (2005) 41:46–52. 10.1016/j.jvs.2004.10.027 15696043

[7] Beck AW Nolan BW De Martino R Yuo TH Tanski WJ Walsh DB. Predicting blood pressure response after renal artery stenting. J Vasc Surg. (2010) 51:380–5. 10.1016/j.jvs.2009.08.088 19939607

[8] Modrall JG Rosero EB Leonard D Timaran CH Anthony T Arko FA. Clinical and kidney morphologic predictors of outcome for renal artery stenting: data to inform patient selection. J Vasc Surg. (2011) 53:1282–9. 10.1016/j.jvs.2010.11.103 21316901

[9] Murphy TP Cooper CJ Matsumoto AH Cutlip DE Pencina KM Jamerson K. Renal artery stent outcomes: effect of baseline blood pressure, stenosis severity, and translesion pressure gradient. J Am Coll Cardiol. (2015) 66:2487–94. 10.1016/j.jacc.2015.09.073 26653621 PMC4819253

[10] Courand PY Dinic M Lorthioir A Bobrie G Grataloup C Denarié N. Resistant hypertension and atherosclerotic renal artery stenosis: effects of angioplasty on ambulatory blood pressure. a retrospective uncontrolled single-center study. Hypertension. (2019) 74:1516–23. 10.1161/HYPERTENSIONAHA.119.13393 31656101

[11] Hall JE Brands MW Shek EW. Central role of the kidney and abnormal fluid volume control in hypertension. J Hum Hypertens. (1996) 10:633–9. 9004086

[12] Ivy JR Bailey MA. Pressure natriuresis and the renal control of arterial blood pressure. J Physiol. (2014) 592:3955–67. 10.1113/jphysiol.2014.271676 25107929 PMC4198007

[13] Cowley AW Roman RJ Fenoy FJ Mattson DL. Effect of renal medullary circulation on arterial pressure. J Hypertens Suppl. (1992) 10:S187–93. 10.1097/00004872-199212000-00021 1291653

[14] Sun YJ Wang SY Ma N Guo FJ Li MP Ai H. An observational study of the effect of baseline renal CBP with contrast-enhanced ultrasound on short-term outcomes of stent implantation for severe renal artery stenosis. Chin J Ultrasonogr. (2021) 30:944–9. 10.3760/cma.j.cn131148-20210430-00301

[15] Jiang XJ Zou YB. Chinese expert consensus for the diagnosis and management of renal artery stenosis. Chin Circul J. (2017) 32:835–44. 10.3969/j.issn.1000-3614.2017.09.002

[16] Ultrasound Branch of Chinese Medical Doctor and Association National Center of Gerontology. Chinese expert consensus on methods and procedures of renal artery contrast-enhanced ultrasound (2021 Edition). Chin J Ultrasonogr. 30:921–6 (2021). 10.3760/cma.j.cn131148-20210827-00605

[17] Ren JH. Standardized thought of examination and operation with contrast-enhanced ultrasound for diagnosis of renal artery stenosis. Natl Med J China. (2020) 100:1281–3. 10.3760/cma.j.cn112137-20200302-00561 32375435

[18] Ren JH Wang SY Ma N Sun YJ Zhang YW Guo FJ. Rationale and study design for one-stop assessment of renal artery stenosis and renal microvascular perfusion with contrast-enhanced ultrasound for patients with suspected renovascular hypertension. Chin Med J. (2019) 132:63–8. 10.1097/CM9.0000000000000002 30628960 PMC6629313

[19] Modrall JG Zhu H Weaver FA. Clinical predictors of blood pressure response after renal artery stenting. J Vasc Surg. (2020) 72:1269–75. 10.1016/j.jvs.2019.12.041 32139312

[20] Silva JA Chan AW White CJ Collins TJ Jenkins JS Reilly JP. Elevated brain natriuretic peptide predicts blood pressure response after stent revascularization in patients with renal artery stenosis. Circulation. (2005) 111:328–33. 10.1161/01.CIR.0000153271.77341.9F 15655135

[21] Jaff MR Bates M Sullivan T Popma J Gao X Zaugg M. Significant reduction in systolic blood pressure following renal artery stenting in patients with uncontrolled hypertension: results from the HERCULES trial. Catheter Cardiovasc Interv. (2012) 80:343–50. 10.1002/ccd.24449 22511402

[22] Weinberg I Keyes MJ Giri J Rogers KR Olin JW White CJ. Blood pressure response to renal artery stenting in 901 patients from five prospective multicenter FDA-approved trials. Catheter Cardiovasc Interv. (2014) 83:603–9. 10.1002/ccd.25263 24307609

[23] Prajapati JS Jain SR Joshi H Shah S Sharma K Sahoo S. Response of blood pressure after percutaneous transluminal renal artery angioplasty and stenting. World J Cardiol. (2013) 5:247–53. 10.4330/wjc.v5.i7.247 23888194 PMC3722422

[24] Rocha-Singh KJ Mishkel GJ Katholi RE Ligon RA Armbruster JA McShane KJ. Clinical predictors of improved long-term blood pressure control after successful stenting of hypertensive patients with obstructive renal artery atherosclerosis. Catheter Cardiovasc Interv. (1999) 47:167–172. 10376497 10.1002/(SICI)1522-726X(199906)47:2<167::AID-CCD7>3.0.CO;2-R

[25] Mishima E Suzuki T Ito S. Selection of Patients for Angioplasty for Treatment of Atherosclerotic Renovascular Disease: Predicting Responsive Patients. Am J Hypertens. (2020) 33:391–401. 10.1093/ajh/hpaa016 31996895

[26] Khan Z Tolia S Sanam K Gholkar G Zughaib M Naik S. Is there still a role for renal artery stenting in the management of renovascular hypertension - A single-center experience and where do we stand? Cardiovasc Revasc Med. (2019) 20:202–6. 10.1016/j.carrev.2018.06.008 29934065

[27] Naghi J Palakodeti S Ang L Reeves R Patel M Mahmud E. Renal frame count: a measure of renal flow that predicts success of renal artery stenting in hypertensive patients. Catheter Cardiovasc Interv. (2015) 86:304–9. 10.1002/ccd.25946 26198066

[28] Mahmud E Smith TW Palakodeti V Zaidi O Ang L Mitchell CR. Renal frame count and renal blush grade: quantitative measures that predict the success of renal stenting in hypertensive patients with renal artery stenosis. JACC Cardiovasc Interv. (2008) 1:286–92. 10.1016/j.jcin.2008.03.012 19463314

[29] Mangiacapra F Trana C Sarno G Davidavicius G Protasiewicz M Muller O. Translesional pressure gradients to predict blood pressure response after renal artery stenting in patients with renovascular hypertension. Circ Cardiovasc Interv. (2010) 3:537–42. 10.1161/CIRCINTERVENTIONS.110.957704 21078879

[30] Leesar MA Varma J Shapira A Fahsah I Raza ST Elghoul Z. Prediction of hypertension improvement after stenting of renal artery stenosis: comparative accuracy of translesional pressure gradients, intravascular ultrasound, and angiography. J Am Coll Cardiol. (2009) 53:2363–71. 10.1016/j.jacc.2009.03.031 19539148

[31] Mitchell JA Subramanian R White CJ Soukas PA Almagor Y Stewart RE. Predicting blood pressure improvement in hypertensive patients after renal artery stent placement: renal fractional flow reserve. Catheter Cardiovasc Interv. (2007) 69:685–9. 10.1002/ccd.21095 17351955

[32] Giroux MF Soulez G Thérasse E Nicolet V Froment D Courteau M. Percutaneous revascularization of the renal arteries: predictors of outcome. J Vasc Interv Radiol. (2000) 11:713–20. 10.1016/S1051-0443(07)61629-7 10877415

[33] Modrall JG Timaran CH Rosero EB Chung J Plummer M Valentine RJ. Longitudinal changes in kidney parenchymal volume associated with renal artery stenting. J Vasc Surg. (2012) 55:774–80. 10.1016/j.jvs.2011.10.026 22264697

